# Attention deficit hyperactivity disorder symptoms and cannabis use after 1 year among students of the i-Share cohort

**DOI:** 10.1192/j.eurpsy.2022.14

**Published:** 2022-03-19

**Authors:** François Arnaud Matthieu Jean, Julie Arsandaux, Ilaria Montagni, Ophélie Collet, Mélina Fatséas, Marc Auriacombe, Josep Antoni Ramos-Quiroga, Sylvana M. Côté, Christophe Tzourio, Cédric Galéra

**Affiliations:** 1 Department of Psychiatry, Dr Jean Eric Techer Hospital, Calais, France; 2 University of Bordeaux, Faculty of Medicine, Bordeaux, France; 3 Bordeaux Population Health Research Center, National Institute of Health and Medical Research (Institut National de la Santé et de la Recherche Médicale—INSERM), Bordeaux, France; 4 Charles Perrens Hospital, Bordeaux, France; 5 Centre Hospitalier Universitaire de Bordeaux (CHU de Bordeaux), Bordeaux, France; 6 National Center for Scientific Research (Centre national de la recherche scientifique—CNRS), Institut de Neurosciences Cognitives et Intégratives d’Aquitaine (INCIA), Bordeaux, France; 7 National Center for Scientific Research (Centre national de la recherche scientifique—CNRS), Addiction Team/SANPSY, Bordeaux, France; 8 Group of Psychiatry, Mental Health and Addiction, Vall d’Hebron Research Institute (VHIR), Barcelona, Spain; 9 Department of Psychiatry, Hospital Universitari Vall d’Hebron, Barcelona, Spain; 10 Biomedical Network Research Centre on Mental Health (CIBERSAM), Instituto de Salud Carlos III, Barcelona, Spain; 11 Department of Psychiatry and Legal Medicine, Universitat Autònoma de Barcelona, Barcelona, Spain; 12 University of Montreal, Quebec, Canada

**Keywords:** ADHD, cannabis, cohort study, epidemiology, students

## Abstract

**Background:**

Cannabis use in university students is associated with academic achievement failure and health issues. The objective of the study was to evaluate the association between attention deficit hyperactivity disorder (ADHD) symptoms and cannabis use after 1 year among students according to previous cannabis use.

**Methods:**

Students in France were recruited from February 2013 to July 2020 in the i-Share cohort. 4,270 participants were included (2,135 who never used cannabis at inclusion and 2,135 who did). The Adult ADHD Self-Report Scale (ASRS) was used to assess ADHD symptoms at inclusion. Cannabis use frequency was evaluated 1 year after inclusion. Multinomial regressions were conducted to assess the association between inclusion ADHD symptoms and cannabis use after 1 year.

**Results:**

Increase in ASRS scores was linked with a greater probability to use cannabis after 1 year and to have a higher cannabis use frequency (once a year—once a month adjusted odds ratio [OR]: 1.24 (1.15–1.34), more than once a month adjusted OR: 1.43 (1.27–1.61)). Among participants who never used cannabis at inclusion, this association disappeared (once a year—once a month adjusted OR: 1.15 (0.95–1.39), more than once a month adjusted OR: 1.16 (0.67–2)) but remained in participants who ever used cannabis at inclusion (once a year—once a month adjusted OR: 1.17 (1.06–1.29), more than once a month adjusted OR: 1.35 (1.18–1.55)).

**Conclusions:**

High levels of ADHD symptoms in students could lead to continued cannabis use rather than new initiations.

## Introduction

Cannabis use during postgraduation from secondary school studies has been associated with a range of adverse outcomes. Notably, current cannabis use is associated with a greater risk of sexually transmitted infections through higher risk-taking behaviors [1]. Cannabis use frequency has also been linked to reduced class attendance, lower academic performance, and less completion of a graduate degree [2–4]. In a Swedish study, heavy cannabis use at 18 years was associated significantly with social welfare assistance (relative risk = 1.38, 95% confidence interval [CI] = 1.19–1.62) and unemployment (relative risk = 1.26, 95% CI = 1.04–1.53) at the age of 40 [5]. In young adults, the risk of depression could be increased by cannabis use [6], and some individuals are exposed to a higher risk of schizophrenia [7] with potential involvement of the catechol-O-methyltransferase (COMT) gene polymorphisms [8].

Risk factors for daily cannabis use in young adults are being male, personal and family stress, impulsivity, low self-esteem, and tobacco smoking [9]. Among risk factors for cannabis use at the individual level, attention deficit hyperactivity disorder (ADHD) could play a specific role. This neuro-developmental disorder, which is characterized by inattention, hyperfocus, hyperactivity, impulsivity, emotional dysregulation, and excessive mind wandering [10], concerns around 7.2% of children and adolescents and 2% of adults [11,12]. ADHD is a known risk factor for substance use [13–17] and substance use disorder [18–22]. An association between ADHD and cannabis use has been suggested by previous studies [13,15,16,18,23]. In their meta-analysis, Lee et al. [19] reported an increased odd of lifetime cannabis use for people with ADHD in childhood (odds ratio [OR] = 2.78, 95% CI = 1.64–4.74) [19]. The meta-analysis of Charach et al. [20] found that ADHD diagnosed in childhood significantly increased the risk of cannabis use disorder during young adulthood (OR = 1.51, 95% CI = 1.02–2.24) [20]. Of note, genetic overlap between ADHD and cannabis use and a causal relationship has been reported [24–26]. Using large meta-analysis of genome-wide association studies, Artigas et al. [24] showed a genetic significant correlation (*R*^2^ = 0.29, *p* = 1.63 × 10^–5^) between ADHD and lifetime cannabis use. In addition, using a two-sample Mendelian randomization approach, Artigas et al. [24] found arguments supporting that ADHD is causal for lifetime cannabis use (OR = 7.9, 95% CI = 3.72, −15.51).

Studies focusing on the association between ADHD and cannabis use have provided controversial results (presence and absence of association) [16,18,27–34]. Prior research in the area is somewhat limited. First, there are few studies on young adults, although they represent the most at-risk population regarding cannabis use. Second, most of the available data for young adults rely on cross-sectional designs, thereby hindering inferences regarding the temporal pattern of the association between ADHD and cannabis use. Third, despite the relevance of psycho-social factors with respect to cannabis use, previous studies lacked a comprehensive adjustment on such factors. Consequently, a residual confusion may impede a proper interpretation of the results. Fourth, most studies until now used clinical samples, which limits the generalization of the results to wider non-clinical populations. Fifth, most of them considered cannabis use and did not explore the influence of use frequency.

We hypothesize that ADHD symptoms at the beginning of adulthood contribute to: (a) subsequent higher frequency of cannabis use, (b) initiate cannabis use in the students without prior history of cannabis use, and (c) maintain the use of cannabis in the students with prior history of cannabis use. A better knowledge of the risk factors of cannabis use could contribute to better targeted public health interventions. University students are a population exposed to cannabis use that could particularly benefit from specific preventive interventions.

The aim of the study was to assess the association between of ADHD symptoms and subsequent cannabis use in a large longitudinal cohort of French students, adjusting on a wide range of sociodemographic, lifestyle, and health confounders, and according to previous cannabis use.

## Methods

### Study design

i-Share is the acronym for Internet-based Students Health Research Enterprise project (www.i-share.fr), an ongoing observational prospective population-based cohort of students in higher education institutions in France, which started in February 2013. The purposes of i-Share are to explore the health, use of addictive substances, and risk behaviors of students. Students were encouraged to participate through active promotion campaigns (via information stands at registration, university emails, lectures, flyers, social media, and newsletters). First, participants filled in a registration form on an online portal and then received an email with their login and password. Second, they had 30 days to validate registration and to complete an online self-administered baseline questionnaire. Between 12 and 24 months after the baseline questionnaire, participants filled in a follow-up self-administered questionnaire online. The participants were not compensated in accordance with French laws.

The i-Share project was approved by the appropriate French national regulatory agencies (Commission nationale de l’informatique et des libertés, CNIL, registration number [DR-2013-019]). The i-Share protocol was submitted to the institutional ethics review board (Comité de protection des personnes, Sud-Ouest et Outre Mer III, CPP). All participants gave their written informed consent to the purpose and the course of the study.

### Population

Inclusion criteria in i-Share were to understand written French, to be 18 years old or above, and to be currently enrolled in a higher education program at inclusion. For this specific study, we used data available up to July 2020. Only students aged between 18 and 30 years old who completed follow-up after 1 year were considered for this work.

### ADHD symptoms

At inclusion, participants completed the Adult ASRS French version 1.1 [35,36]. The ASRS is an exploratory tool for ADHD based on the Diagnostic and Statistical Manual of Mental Disorders—IVth edition (DSM-IV) criteria. It is a short and rapidly completed self-report questionnaire. We calculated the global score by adding the six items, and we dichotomized the ASRS raw score in “low” and “high” levels using the higher strata (score > 18) as defined in the validation study [37]. Both the internal and external validity and the reliability of the ASRS have been demonstrated [35–41].

### Cannabis use

At inclusion, participants were asked: “In your lifetime, have you ever used cannabis?—yes; no; do not wish to reply.” If they answered “do not wish to reply,” their answer was treated as missing data.

After 1 year, participants were asked: “Concerning your use of substances over the last 12 months: did you use cannabis?” If they answered “no,” they were included in the “no” category. If they answered “do not wish to reply,” their answer was treated as missing data. If they answered “yes,” they were asked for frequency of cannabis use by declarative responses among: “every day,” “several times a week,” “once a week,” “several times a month,” “once a month or less,” and “only once.” To obtain a sufficient number of subjects per category, we gathered responses in three new categories: “no,” “≤once/month,” and “> once/month.”

### Covariates

Covariates were chosen based on previous studies [13–16,42–47,48] and on univariate statistical significant associations between ADHD symptoms at baseline and cannabis use 1 year later. At inclusion, using the self-administered online questionnaire, we collected: [1] demographic characteristics: age (continuous, years), sex (male/female) [2]; student-related variables: academic level (first 3 years/fourth year or more of study after the secondary school graduation, in the higher education programs), education degree type for the final year of secondary school (literature, economics, and scientific/technical), social-support allowance (yes/no, having the benefice of financial support by social institutions for the students coming from socially and financially precarious families), job activity (yes/no) [3]; family-related variables: number of siblings (continuous), highest parents’ educational level (graduate or undergraduate of the secondary school studies/postgraduate of the secondary school studies), parental separation (yes/no), parental support during childhood (sparsely/a lot), parent with present or past alcohol issue (no/yes), parent with present or past, depression or anxiety, issue (no/yes) [4]; psychoactive substance uses: current tobacco smoking (yes/no), current alcohol use (less than once a week/once a week or more) [5]; comorbidities: suicidal attempt history (yes/no), depression history (yes/no), obsessive-compulsive disorder history (yes/no), anxious disorder history (yes/no), eating disorder history (yes/no), reading disorder history (yes/no), disability history (yes/no) [6]; and psychiatric symptoms levels: the short version of the Perceived Stress Scale (PSS; continuous) for stress [49,50].

### Statistical methods

For variables presenting missing data, we conducted multiple imputation analyses with the MICE algorithm [51–53] on 50 datasets and 50 iterations. Imputation details, comparisons between available data, complete cases, and imputed data are presented in the Supplementary Material.

First, we described the sociodemographic, lifestyle, and health characteristics, including academic level, psychiatric symptoms, and substance use, in the full sample of participants and according to the history of cannabis use at inclusion. We looked for associations between the cannabis use frequency after 1 year and the other variables using an analysis of variance or the Kruskal–Wallis rank sum test for continuous variables and the Pearson Chi-squared test with Monte Carlo simulated *p*-value based on 10,000 replications for discrete variables. We used a violin chart [54] to illustrate the co-evolution of cannabis use after 1 year and ADHD symptoms according to the history of cannabis use at inclusion.

Second, we conducted multinomial regression model analyses to assess whether baseline ADHD symptoms were linked with cannabis use after 1 year for the full sample and stratified on the history of cannabis use at inclusion. The stratification of analyses on cannabis use history at baseline allowed us to study the initiation (in the students without prior history of cannabis use) and the continuation of cannabis use (in the students with prior history of cannabis use) at the beginning of adulthood. We used imputed data and we have standardized all continuous variables. Variables were introduced sequentially in the model. We first entered standardized ASRS score as predictor only. Next, we added sex and standardized age. Then, we considered all variables associated with cannabis use. We checked collinearity and we selected variables in a data-based approach via the multinomial least absolute shrinkage and selection operator (LASSO) regression [55]. We grouped variables in the lasso regression to keep or to drop a predictor simultaneously in each cumulative link model [56]. Tenfold cross-validation was used to determine the minimal lambda tuning parameter. Lasso regression suggested dropping suicidal attempt history.

Third, we ran sensitivity analyses to test the robustness of the findings [1]: using dichotomized ASRS score as predictor instead of standardized ASRS score [2]; conducting multinomial regressions on complete cases [3]; and conducting weighted multinomial regressions. Weights were computed with calibration on margins with a raking method [57,58] based on reference prevalence of sex and academic level from the 2017 national survey on living conditions of students [59].

Alpha risks were fixed at 5%. All *p*-values were two-tailed. We used OR and their 95% CI to test the independent associations between levels of cannabis use and ADHD symptoms. We used a likelihood ratio test to explore the global association between ADHD symptoms and cannabis use. We performed all analyses using *R* version 4.0.5 [60].

## Results

### Sample description at baseline

**Figure 1.**
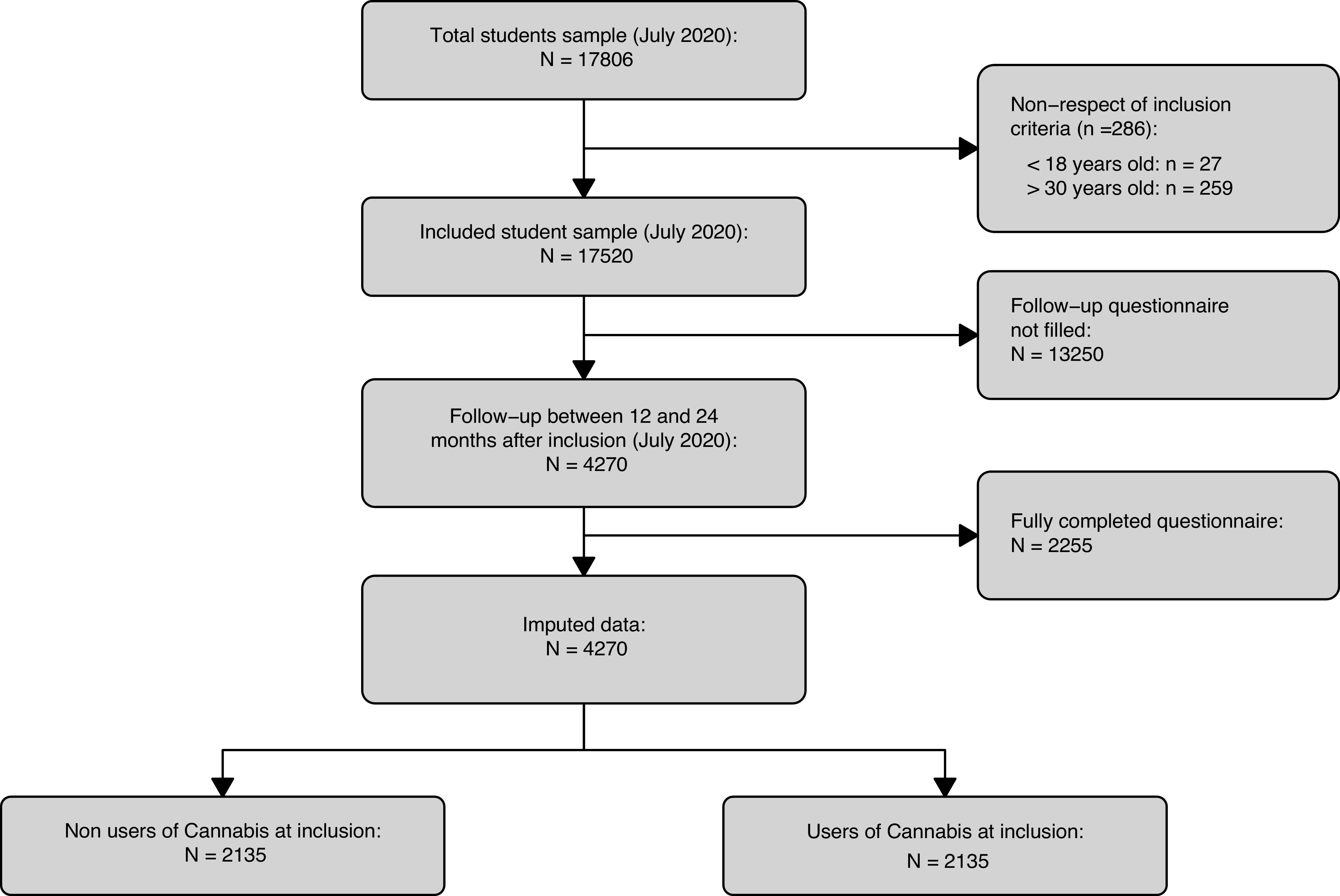
Flowchart of participants. Flowchart shows subjects who never used cannabis at inclusion and who did not complete the follow-up questionnaire. Note vast difference in size between imputed data and complete cases data.

A total of 4,270 participants met the inclusion criteria and completed the follow-up questionnaire (Table 1). Among these participants, 2,135 never used cannabis at inclusion, and 2,135 ever used cannabis at inclusion. The study flowchart is presented in Figure 1.

**Table 1.** Characteristics of sample according to history of cannabis use.

	Total sample (*n* = 4,270)		Cannabis history: no (*n* = 2,135)		Cannabis history: yes (*n* = 2,135)	
	% (*n*) or mean (sd)	p*	% (*n*) or mean (sd)	p*	% (*n*) or mean (sd)	p*
Student variables						
Sex: female	79.7 (3,405)	<0.001	81.9 (1749)	0.172	77.6 (1,656)	<0.001
Age (years)	20.2 (2.2)	0.18	20 (2.1)	0.226	20.5 (2.2)	<0.001
Adult ASRS raw score	10.7 (4)	< 0.001	10.3 (3.9)	0.16	11.1 (4)	<0.001
Dichotomized adult ASRS: high	4.5 (194)	0.002	3.6 (78)	0.003	5.4 (116)	0.056
Substance use						
Cannabis use after 1 year		–		–		–
- no	70.7 (3,018)		93.6 (1,998)		47.8 (1,020)	
- > = 1/year & < = 1/month	22.1 (944)		5.8 (124)		38.4 (820)	
- > 1/month	7.2 (308)		0.6 (13)		13.8 (295)	
Alcohol use: > = 1/week	52.3 (2,232)	<0.001	34.9 (746)	<0.001	69.6 (1,486)	<0.001
Tobacco use: yes	25.3 (1,082)	<0.001	4.6 (99)	<0.001	46 (983)	<0.001
Family variables						
Parental separation	30.8 (1,313)	< 0.001	26 (554)	0.11	35.5 (759)	0.005
Parental support during childhood: a lot	74.8 (3,193)	0.002	76.5 (1,634)	0.747	73 (1559)	0.031
Parent with present or past alcohol issue	10.9 (464)	< 0.001	9.2 (196)	0.213	12.6 (268)	0.004
Parent with present or past depression or anxiety	41.9 (1,788)	0.016	38.5 (822)	0.571	45.2 (966)	0.247

*Note*: *p*-values from ANOVA or Kruskal–Wallis rank sum test, and Pearson Chi-squared test with Monte Carlo simulated *p*-value based on 10,000 replications. *n*, count. p* = *p*-value for the association between the variable and cannabis use after 1 year.

Abbreviations: ASRS, adult ADHD self-report scale; m, mean; sd = standard deviation.

Among participants, there were 79.7% of women (*n* = 3,405), and the mean age was of 20.2 years (standard deviation [SD] = 2.2, range of [18–29]).

Regarding cannabis use 1 year after inclusion for the full sample, 3,018 (70.7%) participants had not used it in the year, 944 (22.1%) participants had used cannabis at least once but less than twice a month, 308 (7.2%) participants had used cannabis more than once a month. The mean level of ADHD symptoms at inclusion was of 10.7 (SD = 4). Further details are provided in supplementary material.

**Figure 2.**
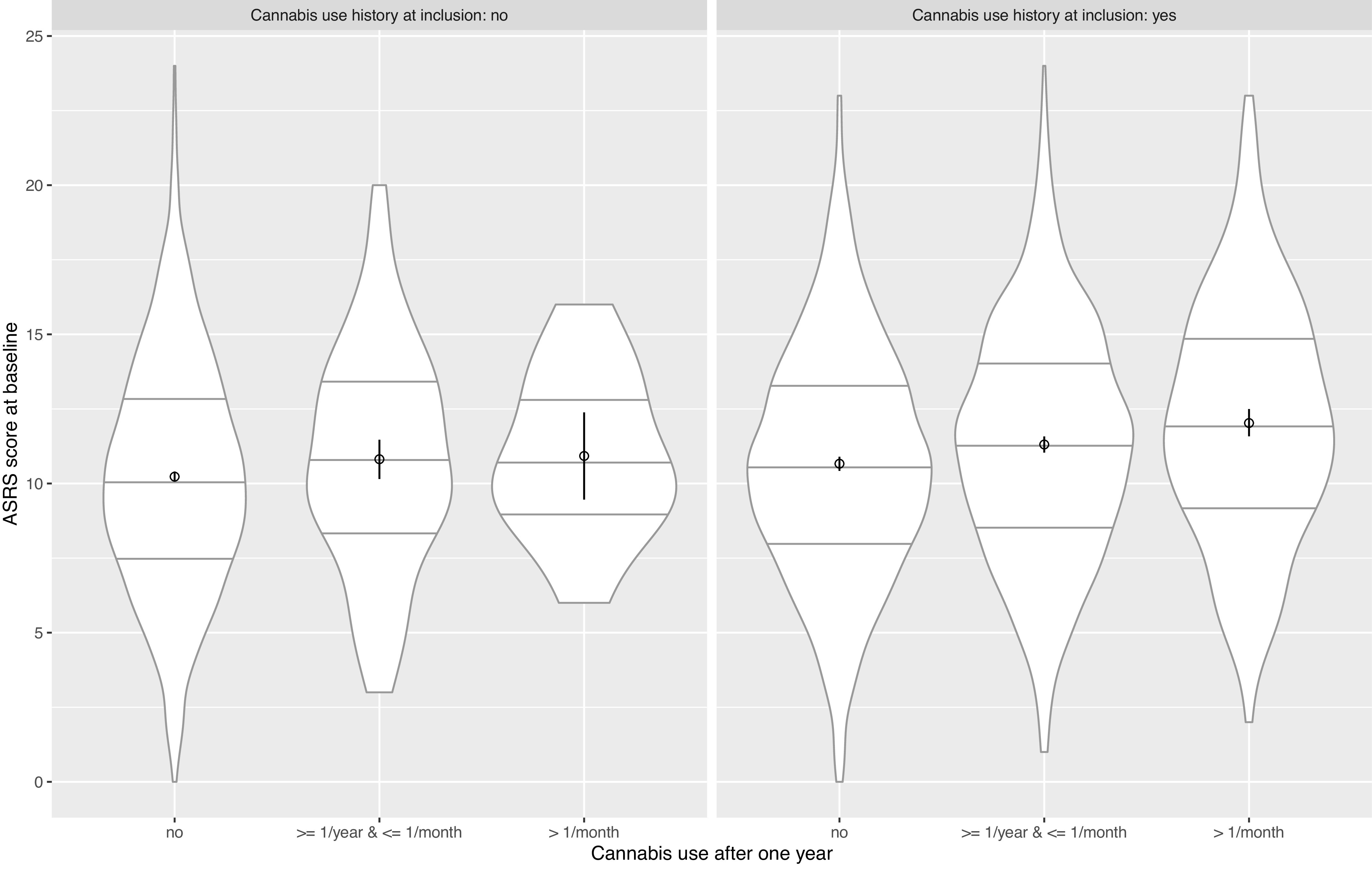
Distribution of ASRS score with mean, and 95% confidence interval of mean among cannabis use levels for nonusers and users of cannabis at baseline. Violin chart with distribution (displayed twice and attached by base), median, quartiles, mean, and 95% confidence interval of mean of ASRS through levels of cannabis use. Note increase in ADHD symptoms with increasing cannabis use among participants who never used cannabis at baseline and among participants who used cannabis at baseline.

The global difference across levels of cannabis use frequency after 1 year for inclusion ASRS raw score was significant for the full sample, for participants who ever used cannabis at inclusion but not for participants who never used cannabis at inclusion (*p* < 0.001, *p* < 0.001, and *p*: 0.16). Figure 2 shows the violin chart [54] figuring the relation between ADHD symptoms at inclusion and cannabis use frequency after 1 year for participants who used and who did not use cannabis at inclusion.

### Multinomial regressions

Table 2 shows the association between ADHD symptoms at inclusion and cannabis use frequency after 1 year. There was an association between ADHD symptoms at inclusion and cannabis use frequency after 1 year in the total sample (*p* < 0.001) with a greater probability to use cannabis between once a year and once a month (adjusted OR: 1.24 (1.15–1.34)) and to use cannabis more than once a month (adjusted OR: 1.43 (1.27–1.61)). Participants who ever used cannabis at inclusion and with high scores of ADHD symptoms on the ASRS were significantly more likely to use cannabis at least once a year but less than twice a month (adjusted OR: 1.24 (1.15–1.34)), and to use cannabis more than once a month (adjusted OR: 1.35 (1.18–1.55)) compared to those who did not use cannabis at all. In participants who never used cannabis at inclusion, high scores of ADHD symptoms on the ASRS were not significantly linked to use cannabis at least once a year but less than twice a month (adjusted OR: 1.15 (0.95–1.39)), and to use cannabis more than once a month (adjusted OR: 1.16 (0.67–2)) compared to those who did not use cannabis at all.

**Table 2.** Associations between ADHD symptoms at inclusion and cannabis use after 1 year (multinomial regression models).

	Total sample (*n* = 4,270)		Cannabis history: no (*n* = 2,135)		Cannabis history: yes (*n* = 2,135)	
	OR (95% CI)	aOR (95% CI)	OR (95% CI)	aOR (95% CI)	OR (95% CI)	aOR (95% CI)
No Cannabis use	1	1	1	1	1	1
Cannabis use: > = 1/year & < = 1/month	1.25 (1.16–1.34)	1.24 (1.15–1.34)	1.16 (0.97–1.39)	1.15 (0.95–1.39)	1.18 (1.08–1.3)	1.17 (1.06–1.29)
Cannabis use: > 1/month	1.5 (1.33–1.68)	1.43 (1.27–1.61)	1.19 (0.69–2.05)	1.16 (0.67–2)	1.42 (1.24–1.61)	1.35 (1.18–1.55)
*p*-value of the LR test on ASRS predictors	< 0.001	< 0.001	0.229	0.302	< 0.001	< 0.001

*Note*: Covariates: age, sex, job activity, parental separation, parental support during childhood, parent with present or past alcohol issue, parent with present or past depression or anxiety issue, depression history, anxious disorder history, eating disorder history.

Abbreviations: aOR, adjusted odds ratio; ASRS, adult ADHD self-report scale; CI, confidence interval; LR, likelihood ratio; OR, odds ratio.

### Sensitivity analysis

Sensitivity analyses showed the same pattern of results. The associations between standardized ASRS scores and cannabis use frequency remained similar and significant, as shown in Table 3 after [1] using dichotomized ASRS [2], selecting uniquely complete cases [3], and weighting on the French student population (respectively for cannabis use less than twice a month: adjusted OR 1.42 (1.01–1.99), adjusted OR: 1.27 (1.15–1.39), adjusted OR: 1.21 (1.12–1.3); and for cannabis use more than once a month: adjusted OR 1.86 (1.17–2.97), adjusted OR: 1.44 (1.24–1.68), adjusted OR: 1.38 (1.22–1.55)).

**Table 3.** Associations between ADHD symptoms at inclusion and cannabis use after 1 year, with dichotomized ASRS, on complete cases and weighted on French students population (multinomial regression models).

	Dichotomized ASRS (*n* = 4,270)	On complete cases (*n* = 2,255)	Weighted on French students (*n* = 4,270)
	aOR (95% CI)	aOR (95% CI)	aOR (95% CI)
No Cannabis use	1	1	1
Cannabis use: > = 1/year & < = 1/month]	1.42 (1.01–1.99)	1.27 (1.15–1.39)	1.21 (1.12–1.3)
Cannabis use: > 1/month	1.86 (1.17–2.97)	1.44 (1.24–1.68)	1.38 (1.22–1.55)
*p*-value of the LR test on ASRS predictors	0.014	< 0.001	< 0.001

*Note*: Covariates: Age, sex, job activity, parental separation, parental support during childhood, parent with present or past alcohol issue, parent with present or past depression or anxiety issue, depression history, anxious disorder history, eating disorder history.

Abbreviations: aOR, adjusted odds ratio; ASRS, adult ADHD self-report scale; CI, confidence interval; LR, likelihood ratio.

## Discussion

### Main findings of study

ADHD symptoms were associated with a higher frequency of cannabis use 1 year later in a population of French students with prior cannabis consumption but not in students with no prior cannabis consumption. This result suggests that ADHD is a potential risk factor for cannabis use continuation but not for initiation during the young-adulthood. Initiation of cannabis use linked to ADHD should have to be done earlier in life. This result extends prior results showing that ADHD is a potential risk factor for cannabis use [13,15,16,18].

### Comparison with other studies and interpretation

Our results complement prior results on the association between cannabis use disorder and use and inattentive and impulsive/hyperactive symptoms. This association is conflicting when considering ADHD symptoms in childhood and cannabis use in adulthood due to the complex role of oppositional behaviors [18,29–34]. Some authors have already reported this association between ADHD symptoms in adolescence and cannabis use in adulthood [34] but not with the focus on no prior cannabis user.

Several hypotheses may explain the prospective link between ADHD and cannabis use. From a genetic perspective, ADHD and substance/cannabis use share a background of common genetic variants [24,61,62]. There are arguments that ADHD is causal for lifetime cannabis use [24]. From a neurobiological perspective, treatment by stimulants reduces the use of substance abuse in persons with ADHD [63,64], consistent with the self-medication hypothesis [65]. Indeed, some studies report results suggesting that the use of cannabis could reduce ADHD symptoms [66–70]. In addition, in students with ADHD, cannabis use is a moderator of ADHD symptoms severity—executive dysfunction relationship and is linked with a reduction of their perceived medications’ side effects [66]. From a cognitive perspective, a deficit in executive functions such as planning and response inhibition is particularly linked with ADHD [71]. Thus, impulsivity and defects in executive functions may play a role in the physio-psycho-pathology of addiction [14]. From a social environmental perspective, we found a link between cannabis use and parental separation, a parent having a past or present alcohol issue, and a lack of parental support during childhood. The ADHD—cannabis use association could be the result of multiple additive and synergistic factors at different levels.

### Strengths and limitations

This work has methodological strengths including the population studied, the large sample size, the longitudinal design, and the analysis of the use frequency. Our samples included young adult students with a large proportion of women. Young adults are a population particularly at risk for the short- and long-term consequences of cannabis use, and longitudinal data on this issue are sparse. Most of the longitudinal studies have focused on childhood or adolescence. The use frequency relationship between cannabis use and ADHD is particularly important and has been used to examine the link between cannabis use and psychotic symptoms [72].

The study also has some limitations. First, since we analyzed only those who completed the follow-up questionnaire, a large number of participants were removed, thus creating a selection bias. However, weighting for demographic characteristics in our sensitivity analysis did not show any differences in results. Second, we selected French students so our findings may not generalize to other nationalities in view of cultural, legal, and genetic variability. Third, we did not adjust for externalizing disorders (conduct disorder or oppositional defiant disorder) since these variables were not measured in the study. It will be interesting in future studies to take into account externalizing disorders since they have been shown to be predictive of cannabis use disorder [73]. Fourth, our results could not be generalized to the full population of young adults since the sample consisted in students uniquely. Students could be different from young adults who did not engage in university education after secondary school graduation. Fifth, it must be acknowledged that in the subsample with prior history of cannabis use it is still possible that ADHD symptoms at baseline could be the result of cannabis use.

### Implications, unanswered questions and future research

Better knowledge of the risk factors of cannabis use could inform public health interventions specifically targeting these risk factors. Students are a population exposed to cannabis, which could be a specific target for such preventive interventions. For instance, a simple screening tool like the ASRS could be made available during online university registration to help students to test themselves and to receive advice. Furthermore, clinicians could advise their patients with ADHD about the specific risk of cannabis use. This advice should be given during adolescence and young adulthood and could form part of a therapeutic patient education program [74]. Randomized controlled trials are needed to determine which interventions can reduce the risk of cannabis use in persons with ADHD. Of course, some questions remain unanswered. The pathway from ADHD symptoms to cannabis use disorder through high cannabis use frequency could be explored via a mediation analysis Cannabis use could have a self-medication purpose to reduce ADHD symptoms and enhance executive functions [66–70], which is relevant regarding the fact that. ADHD is linked with poorer academic achievement [4,75]. Is there a subgroup of ADHD persons using cannabis as self-medication and achieving better academic performance than ADHD persons not using cannabis? Should cannabis be considered for therapeutic use in ADHD persons?

## Conclusions

There is an association between ADHD symptoms and cannabis use in young adulthood. ADHD symptoms are not linked to the initiation of cannabis use in students. High levels of ADHD symptoms represent a potential risk factor for both continuation and an increase in cannabis use in students. Since cannabis use has a negative impact in the short, medium, and long term, ADHD symptoms should be targeted early on.

## Data Availability

The data that support the findings of this study are available from the corresponding author, F.A.M.J. Restrictions apply to the availability of these data, which were under the French law on protection and regulation of data. However, data are available with the permission of the I-share team through a special request.

## Supplementary Materials

To view supplementary material for this article, please visit http://doi.org/10.1192/j.eurpsy.2022.14. click here to view supplementary material
