# Supplementary material for: Attention deficit hyperactivity disorder symptoms and cannabis use after 1 year among students of the i-Share cohort
Source: Eur Psychiatry. 2022 Mar 19;65(1):e25. doi: 10.1192/j.eurpsy.2022.14 (PMC9058443; doi:10.1192/j.eurpsy.2022.14)
Supplement: Supplementary file 1 [file S0924933822000141sup001.docx]

# Supplementary Material

**Table S1: Description of all variables**

|  | **Total sample (n = 4270)** | |  | **Cannabis history: no (n = 2135)** | |  | **Cannabis history: yes (n = 2135)** | |
| --- | --- | --- | --- | --- | --- | --- | --- | --- |
|  | **% (n) or mean (sd)** | **p*** |  | **% (n) or mean (sd)** | **p*** |  | **% (n) or mean (sd)** | **p*** |
| Student variables: |  |  |  |  |  |  |  |  |
| Sex: female | 79.7 (3405) | < 0.001 |  | 81.9 (1749) | 0.172 |  | 77.6 (1656) | < 0.001 |
| Age (years) | 20.2 (2.2) | 0.18 |  | 20 (2.1) | 0.226 |  | 20.5 (2.2) | < 0.001 |
| Academic level: first three years | 73 (3117) | 0.067 |  | 75.9 (1621) | 0.02 |  | 70.1 (1496) | < 0.001 |
| Deprived students' allowance | 39 (1664) | 0.13 |  | 40.8 (872) | 0.448 |  | 37.1 (792) | 0.744 |
| Job activity | 38.4 (1640) | 0.039 |  | 34.1 (728) | 0.601 |  | 42.7 (912) | 0.029 |
| Secondary education degree type: classic | 6.3 (271) | 0.052 |  | 5.5 (117) | - |  | 7.2 (154) | 0.079 |
| Family variables: |  |  |  |  |  |  |  |  |
| Parental separation | 30.8 (1313) | < 0.001 |  | 26 (554) | 0.11 |  | 35.5 (759) | 0.005 |
| Parental support during childhood: a lot | 74.8 (3193) | 0.002 |  | 76.5 (1634) | 0.747 |  | 73 (1559) | 0.031 |
| Parent with present or past alcohol issue | 10.9 (464) | < 0.001 |  | 9.2 (196) | 0.213 |  | 12.6 (268) | 0.004 |
| Parent with present or past depression or anxiety | 41.9 (1788) | 0.016 |  | 38.5 (822) | 0.571 |  | 45.2 (966) | 0.247 |
| Number of siblings | 1.7 (1.1) | 0.566 |  | 1.7 (1.2) | 0.744 |  | 1.6 (1.1) | 0.479 |
| Best parents’ educational level: postgraduate | 57.3 (2446) | 0.193 |  | 54.3 (1160) | 0.204 |  | 60.2 (1286) | 0.668 |
| Substance use: |  |  |  |  |  |  |  |  |
| Cannabis use after one year |  | - |  |  | - |  |  | - |
| - no | 70.7 (3018) |  |  | 93.6 (1998) |  |  | 47.8 (1020) |  |
| - >= 1/year & <= 1/month | 22.1 (944) |  |  | 5.8 (124) |  |  | 38.4 (820) |  |
| - > 1/month | 7.2 (308) |  |  | 0.6 (13) |  |  | 13.8 (295) |  |
| Alcohol use: >= 1/week | 52.3 (2232) | < 0.001 |  | 34.9 (746) | < 0.001 |  | 69.6 (1486) | < 0.001 |
| Tobacco use: yes | 25.3 (1082) | < 0.001 |  | 4.6 (99) | < 0.001 |  | 46 (983) | < 0.001 |
| Psychiatric symptoms and history: |  |  |  |  |  |  |  |  |
| Adult ADHD Self-Report Scale raw score | 10.7 (4) | < 0.001 |  | 10.3 (3.9) | 0.16 |  | 11.1 (4) | < 0.001 |
| Dichotomized Adult ADHD Self-Report Scale: high | 4.5 (194) | 0.002 |  | 3.6 (78) |  |  | 5.4 (116) | 0.056 |
| Perceived Stress Scale raw score | 6.9 (3.2) | 0.21 |  | 6.8 (3.1) | 0.665 |  | 7 (3.2) | 0.387 |
| Suicidal attempts history | 7.7 (328) | 0.001 |  | 6.4 (137) | - |  | 8.9 (191) | 0.033 |
| Depression history | 11 (468) | < 0.001 |  | 8.9 (190) | - |  | 13 (278) | 0.004 |
| Anxious disorder history | 13.4 (572) | < 0.001 |  | 13.1 (279) | - |  | 13.7 (293) | < 0.001 |
| Eating disorder history | 4.5 (192) | < 0.001 |  | 3.9 (84) | - |  | 5.1 (108) | 0.002 |
| Obsessive compulsive disorder history | 1.6 (70) | 0.056 |  | 1.6 (33) | - |  | 1.7 (37) | 0.131 |
| Reading disorder history | 4.4 (187) | 0.626 |  | 5 (107) | - |  | 3.8 (80) | 0.744 |
| History of disability | 2.9 (125) | 0.284 |  | 3.5 (75) | - |  | 2.3 (50) | 0.798 |
| n = count; % = percentage; m = mean; sd = standard deviation, p* = p-value for the association between the variable and cannabis use after one year;   p-values from ANOVA or Kruskal-Wallis rank sum test, and Pearson Chi-squared test with Monte Carlo simulated p-value based on 10000 replications | | | | | | | | |

**Table S2: Description of available cases**

|  | **N Total** | **Missing (%)** | **~~% (n)~~ N (%) or mean (sd)** |
| --- | --- | --- | --- |
| ***Student variables:*** |  |  |  |
| Sex: female | 4270 | 0 (0) | ~~79.7 (3405)~~ 3405 (79.7) |
| Age (years) | 4270 | 0 (0) | 20.2 (2.2) |
| Academic level: first three years | 4233 | 37 (0.9) | ~~73.1 (3093)~~  3093 (73.1) |
| Deprived students' allowance | 4270 | 0 (0) | ~~39 (1664)~~  1664 (39) |
| Job activity | 4270 | 0 (0) | ~~38.4 (1640)~~  1640 (38.4) |
| Secondary education degree type: classic | 4265 | 5 (0.1) | ~~6.3 (269)~~  269 (6.3) |
| ***Family variables:*** |  |  |  |
| Parental separation | 4154 | 116 (2.7) | ~~30.3 (1258)~~  1258 (30.3) |
| Parental support during childhood: a lot | 4223 | 47 (1.1) | ~~74.9 (3162)~~  3162 (74.9) |
| Parent with present or past alcohol issue | 3942 | 328 (7.7) | ~~10.1 (399)~~  399 (10.1) |
| Parent with present or past depression or anxiety | 3516 | 754 (17.7) | ~~40.6 (1428)~~  1428 (40.6) |
| Number of siblings | 4267 | 3 (0.1) | 1.7 (1.1) |
| Best parents’ educational level: postgraduate | 4212 | 58 (1.4) | ~~57.3 (2415)~~  2415 (57.3) |
| ***Substance use:*** |  |  |  |
| Cannabis use after one year | 4239 | 31 (0.7) |  |
| - no |  |  | ~~70.8 (2999)~~  2999 (70.8) |
| - >1/year & <= 1/month |  |  | ~~22.1 (937)~~  937 (22.1) |
| - > 1/month |  |  | ~~4.2 (176)~~  176 (4.2) |
| Alcohol use: >= 1/week | 4270 | 0 (0) | ~~52.3 (2232)~~  2232 (52.3) |
| Tobacco use: yes | 4233 | 37 (0.9) | ~~25.3 (1072)~~  1072 (25.3) |
| ***Psychiatric symptoms and history:*** |  |  |  |
| Adult ADHD Self-Report Scale raw score | 4270 | 0 (0) | 10.7 (4) |
| Perceived Stress Scale raw score | 4270 | 0 (0) | 6.9 (3.2) |
| Suicidal attempts history | 4270 | 0 (0) | ~~7.7 (328)~~  328 (7.7) |
| Depression history | 4270 | 0 (0) | ~~11 (468)~~  468 (11) |
| Anxious disorder history | 4270 | 0 (0) | ~~13.4 (572)~~  572 (13.4) |
| Eating disorder history | 4270 | 0 (0) | ~~4.5 (192)~~  192 (4.5) |
| Obsessive compulsive disorder history | 4270 | 0 (0) | ~~1.6 (70)~~  70 (1.6) |
| Reading disorder history | 4270 | 0 (0) | ~~4.4 (187)~~  187 (4.4) |
| History of disability | 4270 | 0 (0) | ~~2.9 (125)~~  125 (2.9) |
| N: count of available observations, Missing: count of missing observation, n = count; % = percentage; m = mean; sd = standard deviation | | | |

**Table S3: Imputation details**

|  | | **Imputation method** | | **Identifier** | | **Age (years)** | | **Sex: female** | | **Cannabis use after one year** | | | **Alcohol use: >= 1/week** | | **Tobacco use: yes** | | **Cannabis at inclusion** | | **Cannabis history** | | **Adult ADHD Self-Report Scale raw score** | | **Adult ADHD Self-Report Scale inattention score** | | **Adult ADHD Self-Report Scale hyperactivity score** | | | **Perceived Stress Scale raw score** | **Academic level: first three years** | **Secondary education degree type: classic** |
| --- | --- | --- | --- | --- | --- | --- | --- | --- | --- | --- | --- | --- | --- | --- | --- | --- | --- | --- | --- | --- | --- | --- | --- | --- | --- | --- | --- | --- | --- | --- |
| **Identifier** | |  | | 0 | | 1 | | 1 | | 1 | | | 1 | | 1 | | 1 | | 1 | | 1 | | 0 | | 0 | | | 1 | 1 | 1 |
| **Age (years)** | |  | | 0 | | 0 | | 1 | | 1 | | | 1 | | 1 | | 1 | | 1 | | 1 | | 0 | | 0 | | | 1 | 1 | 1 |
| **Sex: female** | |  | | 0 | | 1 | | 0 | | 1 | | | 1 | | 1 | | 1 | | 1 | | 1 | | 0 | | 0 | | | 1 | 1 | 1 |
| **Cannabis use after one year** | | polyreg | | 0 | | 1 | | 1 | | 0 | | | 1 | | 1 | | 1 | | 1 | | 1 | | 0 | | 0 | | | 1 | 1 | 1 |
| **Alcohol use: >= 1/week** | |  | | 0 | | 1 | | 1 | | 1 | | | 0 | | 1 | | 1 | | 1 | | 1 | | 0 | | 0 | | | 1 | 1 | 1 |
| **Tobacco use: yes** | | logreg | | 0 | | 1 | | 1 | | 1 | | | 1 | | 0 | | 1 | | 1 | | 1 | | 0 | | 0 | | | 1 | 1 | 1 |
| **Cannabis at inclusion** | | polyreg | | 0 | | 1 | | 1 | | 1 | | | 1 | | 1 | | 0 | | 1 | | 1 | | 0 | | 0 | | | 1 | 1 | 1 |
| **Cannabis history** | | logreg | | 0 | | 1 | | 1 | | 1 | | | 1 | | 1 | | 1 | | 0 | | 1 | | 0 | | 0 | | | 1 | 1 | 1 |
| **Adult ADHD Self-Report Scale raw score** | |  | | 0 | | 1 | | 1 | | 1 | | | 1 | | 1 | | 1 | | 1 | | 0 | | 0 | | 0 | | | 1 | 1 | 1 |
| **Adult ADHD Self-Report Scale inattention score** | |  | | 0 | | 1 | | 1 | | 1 | | | 1 | | 1 | | 1 | | 1 | | 1 | | 0 | | 0 | | | 1 | 1 | 1 |
| **Adult ADHD Self-Report Scale hyperactivity score** | |  | | 0 | | 1 | | 1 | | 1 | | | 1 | | 1 | | 1 | | 1 | | 1 | | 0 | | 0 | | | 1 | 1 | 1 |
| **Perceived Stress Scale raw score** | |  | | 0 | | 1 | | 1 | | 1 | | | 1 | | 1 | | 1 | | 1 | | 1 | | 0 | | 0 | | | 0 | 1 | 1 |
| **Academic level: first three years** | | logreg | | 0 | | 1 | | 1 | | 1 | | | 1 | | 1 | | 1 | | 1 | | 1 | | 0 | | 0 | | | 1 | 0 | 1 |
| **Secondary education degree type: classic** | | logreg | | 0 | | 1 | | 1 | | 1 | | | 1 | | 1 | | 1 | | 1 | | 1 | | 0 | | 0 | | | 1 | 1 | 0 |
| **Deprived students' allowance** | |  | | 0 | | 1 | | 1 | | 1 | | | 1 | | 1 | | 1 | | 1 | | 1 | | 0 | | 0 | | | 1 | 1 | 1 |
| **Job activity** | |  | | 0 | | 1 | | 1 | | 1 | | | 1 | | 1 | | 1 | | 1 | | 1 | | 0 | | 0 | | | 1 | 1 | 1 |
| **Number of siblings** | | pmm | | 0 | | 1 | | 1 | | 1 | | | 1 | | 1 | | 1 | | 1 | | 1 | | 0 | | 0 | | | 1 | 1 | 1 |
| **Best parents’ educational level: postgraduate** | | logreg | | 0 | | 1 | | 1 | | 1 | | | 1 | | 1 | | 1 | | 1 | | 1 | | 0 | | 0 | | | 1 | 1 | 1 |
| **Parental separation** | | logreg | | 0 | | 1 | | 1 | | 1 | | | 1 | | 1 | | 1 | | 1 | | 1 | | 0 | | 0 | | | 1 | 1 | 1 |
| **Parental support during childhood: a lot** | | logreg | | 0 | | 1 | | 1 | | 1 | | | 1 | | 1 | | 1 | | 1 | | 1 | | 0 | | 0 | | | 1 | 1 | 1 |
| **Parent with present or past alcohol issue** | | logreg | | 0 | | 1 | | 1 | | 1 | | | 1 | | 1 | | 1 | | 1 | | 1 | | 0 | | 0 | | | 1 | 1 | 1 |
| **Parent with present or past depression or anxiety** | | logreg | | 0 | | 1 | | 1 | | 1 | | | 1 | | 1 | | 1 | | 1 | | 1 | | 0 | | 0 | | | 1 | 1 | 1 |
| **Suicidal attempts history** | |  | | 0 | | 1 | | 1 | | 1 | | | 1 | | 1 | | 1 | | 1 | | 1 | | 0 | | 0 | | | 1 | 1 | 1 |
| **Depression history** | |  | | 0 | | 1 | | 1 | | 1 | | | 1 | | 1 | | 1 | | 1 | | 1 | | 0 | | 0 | | | 1 | 1 | 1 |
| **Anxious disorder history** | |  | | 0 | | 1 | | 1 | | 1 | | | 1 | | 1 | | 1 | | 1 | | 1 | | 0 | | 0 | | | 1 | 1 | 1 |
| **Eating disorder history** | |  | | 0 | | 1 | | 1 | | 1 | | | 1 | | 1 | | 1 | | 1 | | 1 | | 0 | | 0 | | | 1 | 1 | 1 |
| **Obsessive compulsive disorder history** | |  | | 0 | | 1 | | 1 | | 1 | | | 1 | | 1 | | 1 | | 1 | | 1 | | 0 | | 0 | | | 1 | 1 | 1 |
| **Reading disorder history** | |  | | 0 | | 1 | | 1 | | 1 | | | 1 | | 1 | | 1 | | 1 | | 1 | | 0 | | 0 | | | 1 | 1 | 1 |
| **History of disability** | |  | | 0 | | 1 | | 1 | | 1 | | | 1 | | 1 | | 1 | | 1 | | 1 | | 0 | | 0 | | | 1 | 1 | 1 |
|  | **Deprived students' allowance** | | **Job activity** | | **Number of siblings** | | **Best parents’ educational level: postgraduate** | | **Parental separation** | | **Parental support during childhood: a lot** | **Parent with present or past alcohol issue** | | **Parent with present or past depression or anxiety** | | **Suicidal attempts history** | | **Depression history** | | **Anxious disorder history** | | **Eating disorder history** | | **Obsessive compulsive disorder history** | | **Reading disorder history** | **History of disability** | | | |
| **Identifier** | 1 | | 1 | | 1 | | 1 | | 1 | | 1 | 1 | | 1 | | 1 | | 1 | | 1 | | 1 | | 1 | | 1 | 1 | | | |
| **Age (years)** | 1 | | 1 | | 1 | | 1 | | 1 | | 1 | 1 | | 1 | | 1 | | 1 | | 1 | | 1 | | 1 | | 1 | 1 | | | |
| **Sex: female** | 1 | | 1 | | 1 | | 1 | | 1 | | 1 | 1 | | 1 | | 1 | | 1 | | 1 | | 1 | | 1 | | 1 | 1 | | | |
| **Cannabis use after one year** | 1 | | 1 | | 1 | | 1 | | 1 | | 1 | 1 | | 1 | | 1 | | 1 | | 1 | | 1 | | 1 | | 1 | 1 | | | |
| **Alcohol use: >= 1/week** | 1 | | 1 | | 1 | | 1 | | 1 | | 1 | 1 | | 1 | | 1 | | 1 | | 1 | | 1 | | 1 | | 1 | 1 | | | |
| **Tobacco use: yes** | 1 | | 1 | | 1 | | 1 | | 1 | | 1 | 1 | | 1 | | 1 | | 1 | | 1 | | 1 | | 1 | | 1 | 1 | | | |
| **Cannabis at inclusion** | 1 | | 1 | | 1 | | 1 | | 1 | | 1 | 1 | | 1 | | 1 | | 1 | | 1 | | 1 | | 1 | | 1 | 1 | | | |
| **Cannabis history** | 1 | | 1 | | 1 | | 1 | | 1 | | 1 | 1 | | 1 | | 1 | | 1 | | 1 | | 1 | | 1 | | 1 | 1 | | | |
| **Adult ADHD Self-Report Scale raw score** | 1 | | 1 | | 1 | | 1 | | 1 | | 1 | 1 | | 1 | | 1 | | 1 | | 1 | | 1 | | 1 | | 1 | 1 | | | |
| **Adult ADHD Self-Report Scale inattention score** | 1 | | 1 | | 1 | | 1 | | 1 | | 1 | 1 | | 1 | | 1 | | 1 | | 1 | | 1 | | 1 | | 1 | 1 | | | |
| **Adult ADHD Self-Report Scale hyperactivity score** | 1 | | 1 | | 1 | | 1 | | 1 | | 1 | 1 | | 1 | | 1 | | 1 | | 1 | | 1 | | 1 | | 1 | 1 | | | |
| **Perceived Stress Scale raw score** | 1 | | 1 | | 1 | | 1 | | 1 | | 1 | 1 | | 1 | | 1 | | 1 | | 1 | | 1 | | 1 | | 1 | 1 | | | |
| **Academic level: first three years** | 1 | | 1 | | 1 | | 1 | | 1 | | 1 | 1 | | 1 | | 1 | | 1 | | 1 | | 1 | | 1 | | 1 | 1 | | | |
| **Secondary education degree type: classic** | 1 | | 1 | | 1 | | 1 | | 1 | | 1 | 1 | | 1 | | 1 | | 1 | | 1 | | 1 | | 1 | | 1 | 1 | | | |
| **Deprived students' allowance** | 0 | | 1 | | 1 | | 1 | | 1 | | 1 | 1 | | 1 | | 1 | | 1 | | 1 | | 1 | | 1 | | 1 | 1 | | | |
| **Job activity** | 1 | | 0 | | 1 | | 1 | | 1 | | 1 | 1 | | 1 | | 1 | | 1 | | 1 | | 1 | | 1 | | 1 | 1 | | | |
| **Number of siblings** | 1 | | 1 | | 0 | | 1 | | 1 | | 1 | 1 | | 1 | | 1 | | 1 | | 1 | | 1 | | 1 | | 1 | 1 | | | |
| **Best parents’ educational level: postgraduate** | 1 | | 1 | | 1 | | 0 | | 1 | | 1 | 1 | | 1 | | 1 | | 1 | | 1 | | 1 | | 1 | | 1 | 1 | | | |
| **Parental separation** | 1 | | 1 | | 1 | | 1 | | 0 | | 1 | 1 | | 1 | | 1 | | 1 | | 1 | | 1 | | 1 | | 1 | 1 | | | |
| **Parental support during childhood: a lot** | 1 | | 1 | | 1 | | 1 | | 1 | | 0 | 1 | | 1 | | 1 | | 1 | | 1 | | 1 | | 1 | | 1 | 1 | | | |
| **Parent with present or past alcohol issue** | 1 | | 1 | | 1 | | 1 | | 1 | | 1 | 0 | | 1 | | 1 | | 1 | | 1 | | 1 | | 1 | | 1 | 1 | | | |
| **Parent with present or past depression or anxiety** | 1 | | 1 | | 1 | | 1 | | 1 | | 1 | 1 | | 0 | | 1 | | 1 | | 1 | | 1 | | 1 | | 1 | 1 | | | |
| **Suicidal attempts history** | 1 | | 1 | | 1 | | 1 | | 1 | | 1 | 1 | | 1 | | 0 | | 1 | | 1 | | 1 | | 1 | | 1 | 1 | | | |
| **Depression history** | 1 | | 1 | | 1 | | 1 | | 1 | | 1 | 1 | | 1 | | 1 | | 0 | | 1 | | 1 | | 1 | | 1 | 1 | | | |
| **Anxious disorder history** | 1 | | 1 | | 1 | | 1 | | 1 | | 1 | 1 | | 1 | | 1 | | 1 | | 0 | | 1 | | 1 | | 1 | 1 | | | |
| **Eating disorder history** | 1 | | 1 | | 1 | | 1 | | 1 | | 1 | 1 | | 1 | | 1 | | 1 | | 1 | | 0 | | 1 | | 1 | 1 | | | |
| **Obsessive compulsive disorder history** | 1 | | 1 | | 1 | | 1 | | 1 | | 1 | 1 | | 1 | | 1 | | 1 | | 1 | | 1 | | 0 | | 1 | 1 | | | |
| **Reading disorder history** | 1 | | 1 | | 1 | | 1 | | 1 | | 1 | 1 | | 1 | | 1 | | 1 | | 1 | | 1 | | 1 | | 0 | 1 | | | |
| **History of disability** | 1 | | 1 | | 1 | | 1 | | 1 | | 1 | 1 | | 1 | | 1 | | 1 | | 1 | | 1 | | 1 | | 1 | 0 | | | |

**Table S4: LASSO regression results**

|  | **Cannabis use after one year: no** | **Cannabis use after one year: [>= 1/year; <= 1/month]** | **Cannabis use after one year: > 1/month** |
| --- | --- | --- | --- |
| **Intercept** | 1.850 | 0.150 | -2.010 |
| **Standardized Age (years)** | 0.100 | -0.060 | -0.040 |
| **Sex: female** | 0.270 | -0.130 | -0.130 |
| **Standardized Adult ADHD Self-Report Scale raw score** | -0.130 | 0.030 | 0.100 |
| **Alcohol use** | -0.800 | 0.160 | 0.640 |
| **Tobacco use** | -1.210 | 0.170 | 1.040 |
| **Job activity** | 0.030 | -0.030 | 0.010 |
| **Parental separation** | -0.100 | -0.080 | 0.180 |
| **Parental support during childhood: a lot** | 0.080 | 0.010 | -0.100 |
| **Parent with present or past alcohol issue** | -0.130 | -0.010 | 0.140 |
| **Parent with present or past depression or anxiety** | 0.060 | -0.020 | -0.040 |
| **Suicidal attempts history** | 0.000 | 0.000 | 0.000 |
| **Depression history** | -0.140 | 0.000 | 0.130 |
| **Anxious disorder history** | 0.110 | -0.220 | 0.110 |
| **Eating disorder history** | -0.250 | -0.050 | 0.300 |
